# Heart rate reduction with esmolol in septic shock: effects on myocardial performance

**DOI:** 10.1186/cc13352

**Published:** 2014-03-17

**Authors:** A Morelli, A D'Egidio, A Orecchioni, E Maraffa, S Romano

**Affiliations:** 1 University of Rome La Sapienza, Rome, Italy; 2 University of Florence, Italy

## Introduction

Clinical study suggests that beta-blockers, by decreasing the heart rate (HR) together with an increase in stroke volume (SV), do not negatively affect cardiac output (CO), allowing an economization of cardiac work and oxygen consumption in patients with septic shock [1]. Whether this hemodynamic profile leads to an amelioration of myocardial performance is still unclear. The objective of the present study was therefore to elucidate whether a reduction in HR with esmolol is associated with an improvement of cardiac efficiency in patients with septic shock who remained tachycardic after hemodynamic optimization.

## Methods

After 24 to 36 hours of initial hemodynamic stabilization, 24 septic shock patients with HR >95 bpm and requiring norepinephrine (NE) to maintain mean arterial pressure (MAP) between 65 and 75 mmHg despite adequate volume resuscitation received a continuous esmolol infusion to maintain the HR between 94 and 80 bpm. NE was titrated to achieve a MAP between 65 and 75 mmHg. To investigate myocardial performance, we simultaneously assessed LV ejection fraction (LVEF), tricuspidal annular plane solid excursion (TAPSE) by echocardiography, the dP/dt MAX and the cardiac cycle efficiency (CCE) both estimated from the arterial pressure waveform. Data were obtained at baseline and after achieving the predefined HR threshold (T1).

## Results

For a MAP between 65 and 75 mmHg, esmolol administration significantly decreased HR (115 ± 10 to 91 ± 7 bpm), NE (0.7 ± 0.4 to 0.5 ± 0.3 μg/kg/minute), and dP/dt MAX (1.1 ± 0.3 to 0.8 ± 0.3 ms/ mmHg). Conversely, TAPSE (15 ± 3 to 20 ± 3 mm), CCE (-0.2 ± 0.4 to -0.03 ± 0.4 units) and SV (37 ± 8 to 42 ± 10 ml) significantly increased at the end of the study period (all *P *< 0.05). CO (4.1 ± 0.8 to 3.9 ± 0.8 l/ minute) and LVEF (46 ± 10 to 48 ± 10%) did not change.

## Conclusion

In patients with established septic shock who remained tachycardic after hemodynamic optimization, titration of esmolol to reduce the HR to a predefined threshold economized cardiac function, resulting in a maintained CO with a lower HR and a higher stroke volume. Such a hemodynamic profile was characterized by an improved cardiac efficiency, as indicated by the decrease in dP/dt MAX associated with an increase in CCE. Finally, echocardiographic data suggest that reducing HR with esmolol positively affects right ventricular function.
